# Oxysterols Increase Inflammation, Lipid Marker Levels and Reflect Accelerated Endothelial Dysfunction in Experimental Animals

**DOI:** 10.1155/2018/2784701

**Published:** 2018-03-11

**Authors:** Tomasz Wielkoszyński, Jolanta Zalejska-Fiolka, Joanna K. Strzelczyk, Aleksander J. Owczarek, Armand Cholewka, Marcin Furmański, Agata Stanek

**Affiliations:** ^1^Department of Biochemistry, School of Medicine with the Division of Dentistry in Zabrze, Medical University of Silesia, Jordana 19, 41-808 Zabrze, Poland; ^2^Department of Medical and Molecular Biology, School of Medicine with the Division of Dentistry in Zabrze, Medical University of Silesia, Jordana 19, 41-808 Zabrze, Poland; ^3^Department of Statistics, Department of Instrumental Analysis, School of Pharmacy with the Division of Laboratory Medicine, Medical University of Silesia, Ostrogórska 30, Sosnowiec 41-209, Poland; ^4^Department of Medical Physics, Chelkowski Institute of Physics, University of Silesia, Uniwersytecka 4, 40-007 Katowice, Poland; ^5^Department of Internal Medicine, Angiology and Physical Medicine, School of Medicine with the Division of Dentistry in Zabrze, Medical University of Silesia, Batorego 15, 41-902 Bytom, Poland

## Abstract

**Objective:**

Oxidized cholesterol derivatives are thought to exert atherogenic effect thus adversely affecting vascular endothelium. The aim of the study was to assess the effect of 5*α*,6*α*-epoxycholesterol on experimentally induced hypercholesterolemia in rabbits, and the levels of homocysteine (HCY), asymmetric dimethylarginine (ADMA), paraoxonase-1 (PON-1), and inflammatory parameters (IL-6, TNF-*α*, CRP).

**Material and methods:**

The rabbits were divided into 3 groups, 8 animals each, and fed with basic fodder (C), basic fodder plus cholesterol (Ch) or basic fodder plus 5*α*,6*α*-epoxycholesterol, and unoxidized cholesterol (ECh). Serum concentrations of studied parameters were determined at 45-day intervals. The study was continued for six months.

**Results:**

We demonstrated that adding 5*α*,6*α*-epoxycholesterol to basic fodder significantly affected lipid status of the experimental animals, increasing total cholesterol and LDL cholesterol levels, as well as HCY and ADMA levels, whilst leaving the PON-1 activity unaffected. Additionally, the ECh group presented with significantly higher concentrations of inflammatory biomarkers (IL-6, TNF-*α*, and CRP). In the Ch group, lower yet significant (as compared to the C group) changes of levels of studied parameters were observed.

**Conclusion:**

Exposure of animals with experimentally induced hypercholesterolemia to 5*α*,6*α*-epoxycholesterol increases dyslipidaemia, endothelial dysfunction, and inflammatory response.

## 1. Introduction

Cardiovascular disease (CVD) is the leading cause of death in Europe and the United States. Heart disease, stroke, and hypertension are currently recognized to be caused, in part, by arterial endothelial dysfunction. Endothelial dysfunction may occur much earlier than the clinical manifestation of cardiovascular diseases [1].

A healthy vascular endothelium remains in a tightly regulated balance between pro and antioxidants, vasodilators and vasoconstrictors, pro and anti-inflammatory molecules, and pro and antithrombotic signals. Dysfunctional endothelium, though, displays prooxidant, vasoconstrictor, proinflammatory, and prothrombotic properties [1–3].

Oxysterols are cholesterol oxidation products formed through enzymatic or autoxidation mechanisms. They may be originally present in food containing animal fat, but they are chiefly generated during food storing and cooking. These compounds show a biochemical reactivity that is one or even two orders of magnitude higher than that of the parent compound. Furthermore, unlike cholesterol, oxysterols are able to permeate through lipophilic membranes [4, 5].

Oxysterols can affect many cellular functions and influence various physiological processes (e.g., cholesterol metabolism, membrane fluidity regulation, and intracellular signaling pathways). They are implicated in a number of pathologies, including type 2 diabetes mellitus, neurodegenerative diseases, inflammatory bowel disease, or degenerative changes within the retina. It has also been suggested that oxysterols may play a role in malignancies such as breast, prostate, colon, and bile duct cancer [5–7]. Furthermore, it has been postulated that oxysterols may play a role in atherosclerosis [8, 9].

In light of such findings, the primary aim of the study was to assess the effect of dietary oxysterols on vascular endothelium.

## 2. Material and Methods

### 2.1. Animals

The protocol was approved by the Bioethical Committee for Animal Experimentation of the Medical University of Silesia in Katowice, Poland (approval number 27/2007, dated April 17th 2007). All animals received humane care in compliance with the 8th edition of the *Guide for the Care and Use of Laboratory Animals* published by the National Institute of Health [10].

Twenty-four male Chinchilla rabbits (b.m. 2870 ± 20 g) were obtained from the Center for Experimental Medicine, Medical University of Silesia in Katowice. The animals were housed individually in stainless steel metabolic cages under a 12-hour light/dark cycle. The rabbits were fed proper fodder (80 g/kg) once a day, allowed unlimited access to water, and weighed at 45-day intervals.

The rabbits were divided into three groups of eight animals each, according to the following scheme:

1. Control group (C): rabbits fed only a basal diet (BD)
2. Cholesterol group (Ch): rabbits fed BD with 0.5% cholesterol, 5% sunflower oil, and 2% porcine lard
3. Oxidized cholesterol group (ECh): rabbits fed BD with 5*α*,6*α*-epoxycholesterol acetate equal to 250 mg free 5*α*,6*α*-epoxycholesterol/kg BD, 0.5% cholesterol, 5% sunflower oil and 2% porcine lard. Daily estimated dose of oxysterol in ECh group was about 10–15 mg/kg

The specific diets for each group were prepared weekly and stored in a freezer at −20°C. The BD was composed of 24% protein, 69% carbohydrate, and 7% fat of the total energy content of the diet. Groups fed BD with cholesterol (Ch group) and 5*α*,6*α*-epoxycholesterol (ECh group) received 19% of energy from proteins, 42% from carbohydrates, and 39% from fat, respectively. The current study was continued for six months.

### 2.2. Synthesis of 5*α*,6*α*-Epoxycholesterol Acetate

5*α*,6*α*-Epoxycholesterol acetate was synthetized from cholesterol acetate (Sigma-Aldrich, USA) by oxidation with m-chloroperoxybenzoic acid (Sigma-Aldrich, USA) as described by McCarthy [11]. Next, oxidation product mixture was purified by column chromatography on silica gel with the use of chloroform-acetone (4 : 1, *v*/*v*) as mobile phase. Fractions containing pure ester were controlled by TLC technique (silica gel plates, solvent as above), pooled, and dried under vacuum.

### 2.3. Blood Sample Collection

At the beginning of experiment (following the acclimatization period) and at 45-day intervals thereafter, 10 mL blood samples were collected from ear veins of each animal to plain and EDTA tubes (Sarstedt, S-Monovette) in a total of six samplings. For serum preparation, samples were allowed to clot and centrifuge (15 min, 1500*g*). The serum was analyzed instantly (lipid parameters and PON-1 activity) or stored deep frozen at −75°C (other assays). Plasma from EDTA tubes was separated immediately after sampling (15 min, 1500 g, 4°C) and stored at −75°C.

### 2.4. Biochemical Analyses

#### 2.4.1. Lipid Profile Parameters Concentrations

Total cholesterol and triacylglycerol concentrations were assayed using a standard enzymatic method (Emapol, Poland). HDL cholesterol was determined using an enzymatic method after precipitation of other lipoproteins with phosphotungstic acid (Emapol, Poland). For LDL cholesterol assay, QUANTOLIP LDL kit (Technoclone, Austria) was used. All analyses were performed using the EM280 biochemical analyzer (Emapol, Poland). Interassay and intra-assay coefficients of variation (CV) were below 3% and 5%, respectively, for all parameters.

#### 2.4.2. Endothelial Dysfunction Markers

*(1) Total Homocysteine Concentration*. Total homocysteine (tHCY) plasma concentration was estimated using the HPLC method with spectrofluorimetric detection according to Kuo et al. [12] and Minniti et al. [13]. HPLC separations were conducted on LC-10ATVP chromatograph (Shimadzu, Japan) equipped with RF-10AXL detector (Shimadzu, Japan) and SUPELCOSIL RP-18 column (4.6 x 150 mm, 5 *μ*m, Supelco, USA). Inter and intra-assay coefficients (CV) of variation were 7.7% and 11.2%, respectively.

*(2) Asymmetric Dimethylarginine Concentration*. Asymmetric dimethylarginine (ADMA) plasma concentration was determined using the HPLC method as described previously [14, 15]. Assays were performed on Nucleosil Phenyl column (25 × 4.6 mm; 7 *μ*m; Supelco, USA) and Shimadzu chromatograph with spectrofluorimetric detector (as described above). Inter and intra-assay coefficients (CV) of variation were 7.2% and 10.9%, respectively.

*(3) Paraoxonase-1 Activity*. Paraoxonase-1 (PON-1) serum activity was assayed using the kinetic method with paraoxon (*o,o*-diethyl-*o-(p*-nitrophenyl)-phosphate; Sigma, USA) as a substrate [16]. Determinations were performed at 37°C on TECHNICON RA-XT™ analyzer (Technicon Instruments Corporation, USA). For cholinesterase inactivation, physostigmine salicylate (eserine) was added to serum samples prior to the assay. One unit (1 IU) of PON-1 is the amount of enzyme sufficient to decompose 1 micromole of substrate per minute under testing conditions. Inter and intra-assay coefficients (CV) of variation were 2.6% and 4.4%, respectively.

#### 2.4.3. Inflammatory Markers

*(1) C-Reactive Protein Concentration*. C-reactive protein (CRP) serum concentration was determined using ELISA assay with use the immunoaffinity purified, hen antirabbit CRP antibody as a capture antibody, rabbit CRP reference serum as a standard, and biotinylated CCRP-15A-Z antibody (all reagents were from Immunology Consultants Laboratory Inc., USA) along with streptavidin-horseradish peroxidase conjugate (DakoCytomation, Denmark) for the immunocomplex detection. Inter and intra-assay coefficients of variation were 5% and 7.6%, respectively.

*(2) Tumor Necrosis Factor α Concentration*. The concentration of rabbit tumor necrosis factor *α* (TNF-*α*) in serum was measured using ELISA method with goat antirabbit TNF-*α* antibody as a capture antibody, biotinylated, monoclonal antirabbit TNF-*α* antibody (both from BD PharMingen, USA), and streptavidin-horseradish peroxidase conjugate (DakoCytomation, Denmark) as a tracer. The assay was performed according to the manufacturer's instruction and calibrated with the use of rabbit TNF-*α* (BD PharMingen, USA). Results were presented as pg of TNF-*α* per mL of serum [pg/mL]. Inter and intra-assay coefficients of variation were 6.4% and 8.9%, respectively.

### 2.5. Statistical Analyses

Statistical analysis was performed using STATISTICA 10.0 PL (StatSoft, Poland, Cracow) and StataSE 12.0 (StataCorp LP, TX, U.S.) bundles and R software. *p* value below 0.05 was considered as statistically significant. All tests were two tailed. Imputations were not done for missing data. Nominal and ordinal data were expressed as percentages, whilst interval data were expressed as mean value ± standard deviation if normally distributed or as median/interquartile range if the distribution was skewed or nonnormal. Distribution of variables was evaluated by the Shapiro–Wilk test and homogeneity of variances was assessed using the Levene test. The comparisons were made using one-way parametric ANOVA with Tukey's posthoc test and one-way repeated measures ANOVA with contrast analysis as a posthoc test.

## 3. Results

### 3.1. Animal Body Weight

**Figure 1:**
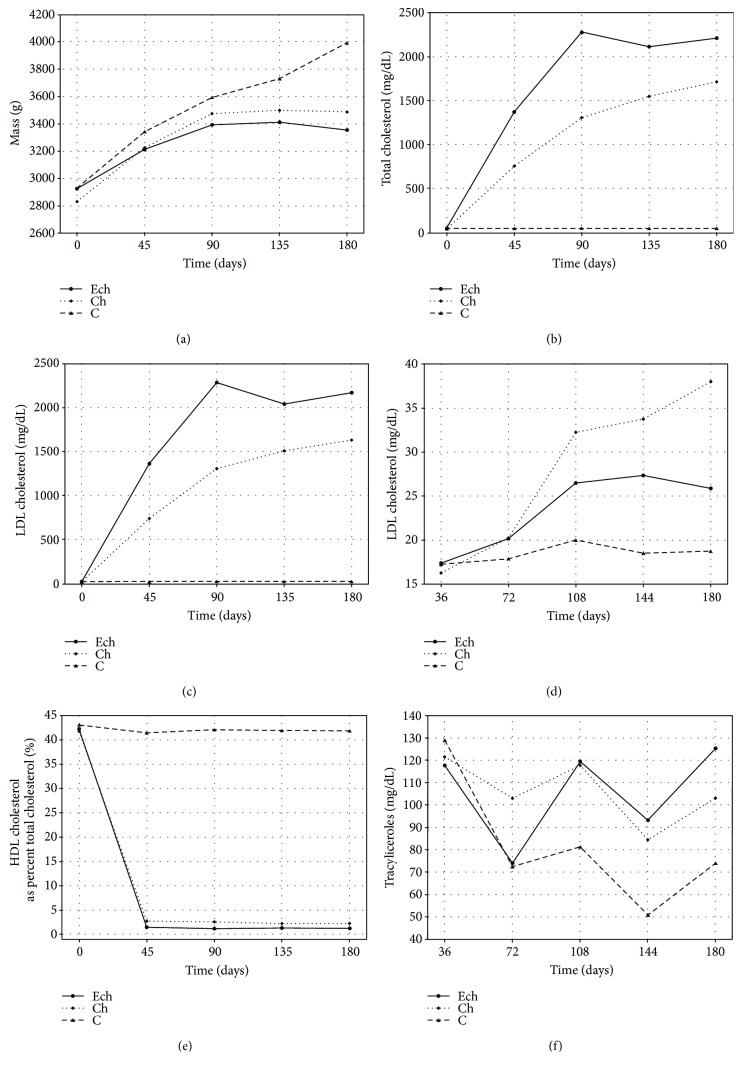
Time's profile of body weight and concentrations of total cholesterol, LDL cholesterol, HDL cholesterol, HDL cholesterol as a percentage of total cholesterol (% HDL), and triacylglycerols in rabbits exposed to 5*α*,6*α*-epoxycholesterol and cholesterol (ECh group), cholesterol (Ch group), and fed with basal diet (C group).

The analysis of animal body weight during the experiment demonstrated a statistically significant growth inhibition in a group fed with 5*α*,6*α*-epoxycholesterol acetate (ECh group) as compared to the control group. There were no significant profile differences between the C and Ch groups.  Figure 1 shows measurement results, and Table 1 shows the result of statistical analyses for the studied variables.

### 3.2. Lipid Profile Parameters

Changes in total cholesterol and LDL cholesterol during the experimental exposure to oxysterols and cholesterol demonstrated significant differences in concentration increase rates between the groups of experimental animals. The fastest concentration increase was seen in the group fed with both 5*α*,6*α*-epoxycholesterol and cholesterol (ECh group). Total cholesterol and LDL cholesterol levels in this group reached the plateau on day 90 and remained unchanged thereafter. In a group fed with BD and cholesterol only (Ch group), the concentration of the markers in question increased at a slower rate to eventually settle at a lower level. The total cholesterol and LDL cholesterol concentration profiles in ECh group differed significantly from those of the Ch group (see Figure 1 and Table 1). Together, ECh and Ch groups had significantly higher levels of total cholesterol and LDL cholesterol as compared to the control group (C), where there was no change in these parameters throughout the entire experiment.

The HDL cholesterol levels tended to increase in both cholesterol-fed groups (ECh and Ch groups). The highest increase of HDL cholesterol levels was noted in a group fed with cholesterol-rich diet without oxysterols (Ch group). Furthermore, there was a significant difference in HDL cholesterol profile between ECh and Ch groups. The analysis of HDL cholesterol concentration as a percentage of total cholesterol did not demonstrate changes between the group exposed to oxysterols and cholesterol versus the group exposed to native cholesterol only (Figure 1 and Table 1).

Triacylglycerol levels varied in a nonspecific way with no significant differences between the groups. There was a significant variability between the individual time points (collections). In a control group, triacylglycerol level at the end of the experiment was significantly lower than the baseline value, whereas in the two remaining groups, there were no significant differences between the baseline and final levels (Figure 1 and Table 1).

### 3.3. Endothelial Dysfunction Markers

**Figure 2:**
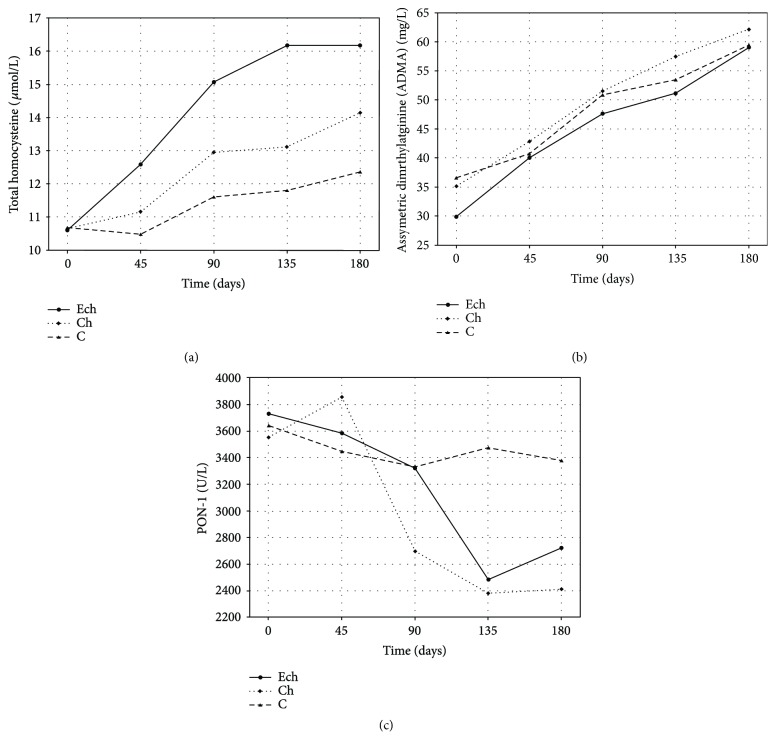
Time's profile of concentrations of total homocysteine (tHCY), asymmetric dimethylarginine (ADMA), and paraoxonase-1 (PON-1) activity in rabbits exposed to 5*α*,6*α*-epoxycholesterol and cholesterol (ECh group), cholesterol (Ch group), and fed with basal diet (C group).

The analysis of changes in total plasma tHCY concentration in rabbits demonstrated an increase tendency in a control group and a marked significant increase in both cholesterol-fed groups. The highest increase was observed in a group exposed additionally to 5*α*,6*α*-epoxycholesterol. The tHCY levels in this group differed significantly from the only cholesterol-fed group (Ch group) or the control group (Figure 2, Table 1).

Similar observations were made for plasma ADMA in rabbits. The highest rate of ADMA concentration increase was seen in ECh (Figure 2, Table 1).

Serum PON-1 activity in rabbits dropped significantly as a result of exposure to dietary cholesterol. However, there was no additional modulation of its activity by oxysterols, as PON-1 levels decreased in the same manner in both ECh group, exposed to oxycholesterol, and Ch group, exposed to cholesterol. Figure 2 shows PON-1 activity at subsequent time points, and Table 1 shows statistical analysis of these results.

### 3.4. Inflammatory Markers

**Figure 3:**
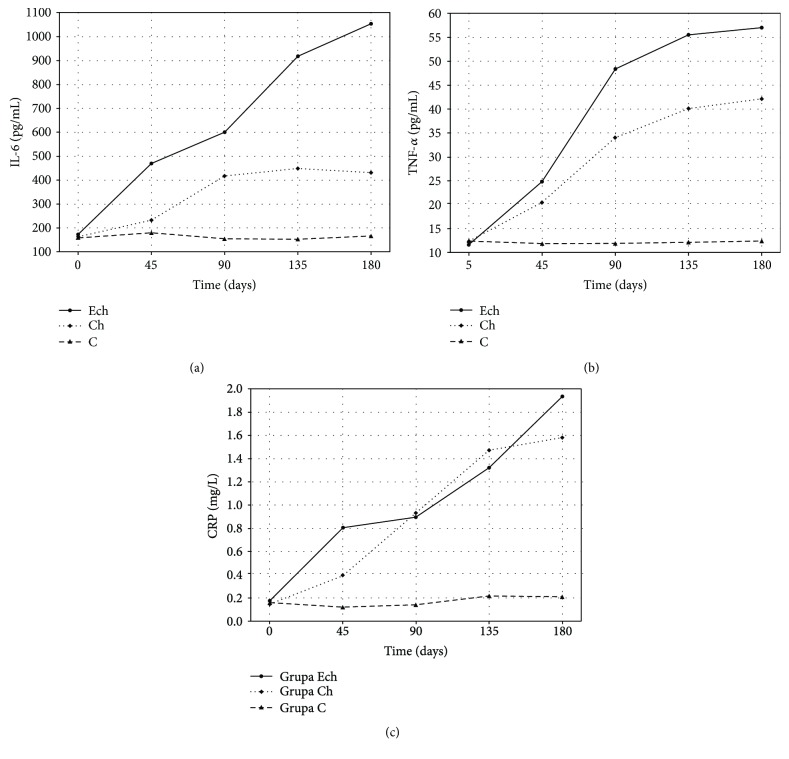
Time's profile of concentrations of IL-6, TNF-*α*, and C-reactive protein (CRP) in rabbits exposed to 5*α*,6*α*-epoxycholesterol and cholesterol (ECh group), cholesterol (Ch group), and fed with basal diet (C group).

The analysis of changes in inflammatory marker concentrations during experimental rabbit exposure to cholesterol and its epoxy derivatives demonstrated activation of acute phase response in studied animals (see Figure 3 and Table 1). Changes in IL-6 levels demonstrated a significant increase in its production following an exposure to 5*α*,6*α*-epoxycholesterol as compared to exposure to native cholesterol or basal diet. Its increase rate and the ultimate level achieved in month 6 in the ECh group were significantly higher than in the Ch and C groups, respectively.

A similar pattern was observed for the serum TNF-*α* concentration during the experimental exposure to epoxycholesterol. The highest concentrations were achieved in animals exposed to 5*α*,6*α*-epoxycholesterol and cholesterol-rich fodder. The TNF-*α* profile in the ECh group significantly differed to the one of the control group and the group receiving cholesterol-rich fodder (Ch). Although the cytokine in question was also biosynthesized in the Ch group, the increase rate and the ultimate TNF-*α* level were significantly lower than in the ECh group.

The serum CRP concentration increased in a similar manner in both groups exposed to cholesterol. The CRP profiles of ECh and Ch groups were significantly different to the one of the control group. There was no significant difference in CRP profiles between the ECh and Ch groups (see Figure 3 and Table 1).

## 4. Discussion

The results achieved during the current study demonstrated changes in the lipid parameters, endothelial dysfunction markers (tHCY, ADMA, and PON-1), and selected inflammatory markers in rabbits fed with cholesterol-rich diet with added oxidized cholesterol derivatives for 6 months. The concentrations of all these markers were assessed several times at 45-day interval, which undoubtedly adds value to the current study, as the experiments reported so far provided only baseline and end concentrations of these parameters. Having reviewed the available literature, we failed to identify a study to assess changes in serum biochemical markers during exposure to cholesterol-rich diet and oxysterols.

In our research, we found total cholesterol and LDL cholesterol levels in rabbits fed with cholesterol-rich diet increased several dozenfold, with the highest increase observed in a group of animals fed with both cholesterol and 5*α*,6*α*-epoxycholesterols. Both total cholesterol and LDL cholesterol levels in this group of animals were significantly higher than in those fed with unoxidized cholesterol. Therefore, adding 5*α*,6*α*-epoxycholesterols to the fodder increased plasma levels of total cholesterol and LDL cholesterol. The study by Mahfouz et al. [17] did not confirm that replacing native cholesterol with autooxidized cholesterol leads to changes in the plasma lipid profile. The mechanisms leading to these observable changes in plasma cholesterol levels are difficult to explain, especially that most published data support inhibition by oxysterols of HMG-CoA reductase activity, which should be reflected in decreased biosynthesis of endogenous cholesterol. On the other hand, it can be hypothesized that endogenous cholesterol in plasma of rabbits with experimentally induced hypercholesterolemia constitutes only a small share of total amount of cholesterol, so it is bioavailability (intestinal absorption and excretion) of exogenous, dietary cholesterol, which mainly affects the blood concentration. Another possible explanation is that 5*α*,6*α*-epoxycholesterols exert their effect at the stage of intestinal absorption, as with the unchanged plasma levels of biliary acids (unpublished data), slowing of cholesterol metabolite elimination appears less likely mechanism contributing to increasing hypercholesterolaemia [18].

The analysis of changes in HDL cholesterol levels in rabbit serum demonstrated that absolute concentrations of this fraction increased heterogeneously in the groups exposed to cholesterol, with the highest increase observed in the Ch group. The difference between the ECh and Ch groups, though, was nonsignificant when the comparison was made between HDL cholesterol levels expressed as a percentage of total cholesterol in a given group. This finding indicates the increased HDL production in the liver in response to increased dietary cholesterol intake, which is in keeping with published data [19].

Analysing the results of tHCY and ADMA assays in a rabbit model, it becomes clear that the exposure to oxidized cholesterol derivatives and cholesterol leads to their gradual plasma level elevation and increases endothelial dysfunction as compared to the controls. The plasma levels of both tHCY and ADMA were elevated following the exposure to oxysterols and cholesterol. However, exposure to cholesterol only also resulted in a significant elevation of these biomarkers. Significant elevation of HCY and ADMA levels in a group fed with fodder containing 5*α*,6*α*-epoxycholesterols, as compared to the groups C and Ch, indirectly confirms additional effect of epoxycholesterols on tHCY and ADMA levels. The HCY and ADMA levels in the ECh group were significantly higher than in the Ch group. In the ECh group, plasma ADMA levels tended to start increasing earlier and more markedly, which can be attributed to the angiotoxic effect of oxysterols on vascular endothelium or abnormal renal ADMA elimination.

The analysis of IL-6 concentrations in our experimental animals seems to suggest that IL-6 biosynthesis is regulated by exogenous epoxycholesterols, as IL-6 concentrations increased significantly faster to settle at a higher level in animals exposed to oxycholesterol as compared to the groups Ch and C. In the group Ch, the IL-6 level in month 6 was twice as low as in the ECh group. In the control group, it remained unchanged throughout the experiment. The above findings are comparable to those published previously [20–22]. However, the values reported in individual papers tend to differ markedly, potentially due to different assay methods used. Due to the well-established role of IL-6 in promoting atherosclerosis in humans, its increased biosynthesis seen in a rabbit model (particularly marked in a group exposed to oxysterols) appears to be a significant biomarker suggestive of chronic vascular wall inflammation.

In our experiment, the TNF-*α* concentration in rabbits exposed to unoxidized cholesterol significantly increased over the first four months to stabilise over the subsequent two months. In the group fed additionally with 5*α*,6*α*-epoxycholesterol, the rate and extent of increase were significantly higher with higher TNF-*α* levels in month 6.

Rabbits with experimentally induced hypercholesterolemia presented with elevated levels of TNF-*α*, IL-6, IL-1, and selected endothelial dysfunction markers, as it was seen in a rat model [23]. Direct exposure of HUVEC cells to 7-keto-, 7*β*-hydroxycholesterol, and 7*α*-hydroxycholesterol resulted in an increased TNF-*α* production [24]. This can explain elevated TNF-*α* levels in animals exposed to oxysterols. However, the available literature lacks data on the effect of 5*α*,6*α*-epoxycholesterol on biosynthesis of inflammatory cytokines.

The elevated levels of CRP in animals with experimentally induced hypercholesterolemia, demonstrated in the current study, are consistent with the available published data [20, 23, 25]. Additional intake of dietary 5,6-epoxycholesterol did not affect CRP levels. There is data to confirm extrahepatic origin of CRP in rabbits with experimentally induced hypercholesterolemia, as its synthesis in adipocytes was shown to be inhibited by administering atorvastatin [26]. Perhaps, then, animal adipose tissue constitutes an alternative source of this protein in the plasma, which is true for some proinflammatory cytokines, such as TNF-*α*. This hypothesis, though, appears unlikely due to decreased weight gain observed in animals exposed to oxysterols as compared to animals in the groups C and Ch.

The current study has some limitations. Extrapolating the findings of experimental research in animal models to the risk of oxysterol intake by humans, it should be noted that the intake of cholesterol derivatives in experimental animals ranged between 2.5 to approx. 10 mg/kg per day [27, 28], which translates into the intake of 175–700 mg of oxysterols per day in an adult and amounts to at least 100% of typical cholesterol daily intake. Therefore, it is highly unlikely that a diet of a modern individual can comply with these assumptions. Another issue is interspecies differences between experimental animals (usually rabbits, rodents, or birds) and humans. A limitation of the current study is also a relatively low number of experimental animals in each group, which was guided by ethical considerations relevant to animal research experiments.

## 5. Conclusions

Based on our findings, we conclude that having been absorbed from the gastrointestinal tract and incorporated in lipoprotein structures, oxidized cholesterol derivatives exert cytotoxic effect on vascular endothelial cells, causing endothelial dysfunction, the severity of which depends on the duration of exposure. Combined administration of oxysterols and cholesterol is likely to increase their angiotoxic effect. At the same time, the inflammatory response and dyslipidaemia increase in severity.

## Figures and Tables

**Table 1:** Results of ANOVA analysis of parameters between groups and inside each group (between first and last sampling).

Parameter	Body mass	Total cholesterol	LDL cholesterol	HDL cholesterol	HDL cholesterol as percent of total cholesterol	Triacylglycerols	tHCY	ADMA	PON-1	IL-6	TNF-*α*	CRP
Differences between groups for all sampling (*p values)*												
C–ECh	<0.05	<0.001	<0.001	<0.01	<0.001	<0.05	<0.001	<0.001	NS	<0.001	<0.001	<0.001
C–Ch	NS	<0.001	<0.001	<0.001	<0.001	<0.05	<0.05	<0001	<0.01	<0.001	<0.001	<0.001
ECh–Ch	NS	<0.001	<0.001	<0.01	NS	NS	<0.01	<0.001	NS	<0.001	<0.001	NS

Differences inside each group between 0 day and 180 day (*p values)*												
Ech	<0.001	<0.001	<0.001	<0.01	<0.001	NS	<0.001	<0.001	<0.001	<0.001	<0.001	<0.001
Ch	<0.001	<0.001	<0.001	<0.001	<0.001	NS	<0.001	<0.001	<0.001	<0.05	<0.001	<0.001
C	<0.001	NS	NS	NS	NS	<0.001	NS	NS	NS	NS	NS	NS

NS: nonstatistically significant.
